# Comparative Tolerance Levels of Maize Landraces and a Hybrid to Natural Infestation of Fall Armyworm

**DOI:** 10.3390/insects13070651

**Published:** 2022-07-19

**Authors:** Andreísa Fabri Lima, Julio Bernal, Maria Gabriela Silva Venâncio, Bruno Henrique Sardinha de Souza, Geraldo Andrade Carvalho

**Affiliations:** 1 Department of Entomology, Lavras Federal University (UFLA), Lavras 37200-900, MG, Brazil; andreisaflima@gmail.com (A.F.L.); maria.svenancio@outlook.com (M.G.S.V.); gacarval@ufla.br (G.A.C.); 2 Department of Entomology, Texas A&M University, College Station, TX 77840, USA

**Keywords:** host plant resistance, *Spodoptera frugiperda*, compensation, overcompensation, plant defense

## Abstract

**Simple Summary:**

Exploiting the tolerance of plants against herbivorous insects is a viable pest management alternative, especially where conventional controls are ineffective. For example, due to the inefficacy of currently adopted practices, new strategies and methods are needed for *Spodoptera frugiperda* management in maize. This study evaluated the tolerance levels of maize landraces and a conventional hybrid under natural infestation of *S. frugiperda*. We found promising sources of tolerance among the landraces, evident as tolerance indices that varied across the landraces and hybrid we evaluated.

**Abstract:**

Insect pests such as *Spodoptera frugiperda* cause significant losses to maize (*Zea mays mays*). Control of *S. frugiperda* is difficult, but the use of insect resistant cultivars, including tolerant cultivars, is a promising alternative, and landraces are a potential source of insect resistance. This study investigated tolerance to *S. frugiperda* in five Brazilian landraces, Amarelão, Aztequinha, Branco Antigo, Palha Roxa, and São Pedro, in relation to one conventional (non-Bt) hybrid, BM207, under field conditions. We assessed tolerance as the ratio of insecticide-free to insecticide-protected plants for plant height, stem diameter, and leaf chlorophyll content at two plant stages. Tolerance ratios varied across the maize genotypes, but inconsistently across plant variables, and cluster analysis revealed three groups based on tolerance ratios. A first group contained genotypes similarly tolerant to *S. frugiperda*, BM207, Palha Roxa, São Pedro, and Aztequinha, while the second and third groups each contained single genotypes, Amarelão, and Branco Antigo, which were considered not tolerant. Overall, the landraces Palha Roxa, São Pedro, and Aztequinha compared favorably to BM207 in terms of tolerance, and therefore may be valuable for management of this pest, and as germplasm sources to improve tolerance in other cultivars.

## 1. Introduction

Maize (*Zea mays mays* L.) crops are constantly affected by abiotic and biotic stresses, including attack of pest insects, which are the main biotic stressors impacting crop yield [1,2]. Fall armyworm (hereafter FAW), *Spodoptera frugiperda* (J. E. Smith) (Lepidoptera: Noctuidae), is one of the most important insect pests affecting maize crops in the Americas [3,4,5], mainly due to the polyphagous habit of the species [5,6,7]. In maize, FAW larvae preferentially feed on young leaves, compromising plant growth [8].

FAW is native to the tropical and subtropical Americas. However, due to its capacity for long-distance flight, and broad environmental adaptation [9], FAW has become an invasive pest in Africa [10,11], and more recently in India [12,13] and China [9]. Upon its occurrence in new areas, control methods need to be integrated for effective management of this pest [14]. Control of FAW is usually carried out with insecticide applications and genetically modified cultivars and hybrids expressing toxic proteins of the bacterium *Bacillus thuringiensis* (*Bt*) in crops and countries where they are available. However, FAW has shown resistance to maize *Bt* hybrids [15,16,17,18], as well as to insecticides [19,20,21,22]. Overall, resistance of FAW populations to the main control methods are a challenge for the effective management of this pest, requiring new strategies to ensure the productivity of affected crops, such as maize. 

Host plant resistance (HPR) is a fundamental component of integrated pest management (IPM) programs. HPR comprises antibiosis, through plant traits that affect pest survival, development, and reproduction; antixenosis, through traits that affect pest colonization; and tolerance, through traits that allow plants to withstand pest injury without substantially compromising productivity [23,24]. All three forms of HPR can be incorporated in crop cultivars and hybrids through traditional breeding or genetic engineering [25]. Tolerance may play important roles in crop protection, especially in cases where insect pests do not transmit pathogens [26], or where resistance to pests is low [27]. Tolerance does not directly affect pest insects, thus it is presumed to not contribute to the selection of resistant biotypes [28,29]. Furthermore, tolerant genotypes can sustain greater pest injury before requiring insecticide applications [29].

Tolerance is associated with greater efficiency in plant photosynthetic activity, better use of stored reserves, and appropriate phenological changes [26,30]. These mechanisms may generate different levels of tolerance, whether compensation or overcompensation for lost tissues, or undercompensation, i.e., non-tolerance [31]. Overcompensation can occur for vegetative and/or reproductive plant tissues, and may vary according to the plant genotype [32,33], and it can be exploited in agriculture due to the direct impact on crop productivity [34]. For instance, potato (*Solanum tuberosum* L.) plants increase their productivity when injured by *Tecia solanivora* (Povolny) (Lepidoptera: Gelechiidae) larvae, and yield can increase by up to 100% when 10% of tubers are damaged by specialist herbivores [35,36]. In maize, tolerance traits are diverse, and include mechanisms contributing to greater root system growth and biomass in the case of *Diabrotica virgifera virgifera* LeConte (Coleoptera: Chrysomelidae) larvae [37,38], or compensatory shoot growth due to allocation of photoassimilates [39]. Tolerance to *Diabrotica speciosa* (Germar) (Coleoptera: Chrysomelidae) in the Brazilian maize landrace Azteca is associated with greater number of photosynthetic pigments [40].

Maize landraces are open-pollinated varieties with broad genetic bases that were selected by the environment and farmers over many generations, and that maintain moderate stress resistance and yield characteristics [41]. They carry high genetic diversity, so are valuable genetic resources for breeding programs, particularly breeding directed at improving agronomic parameters and food security [42,43]. Several studies reported maize landraces with resistance to arthropod pests [44,45,46,47,48,49]. However, tolerance traits are poorly studied and widespread, despite their potential use in IPM and genetic breeding programs, so they merit additional research [29,40].

Given the growing demand for effective management strategies for FAW, especially in developing countries, we evaluated five landraces and one commercial hybrid for their tolerance to this pest. Specifically, we conducted field experiments in which tolerance to FAW in the Brazilian maize landraces Amarelão, Aztequinha, Branco Antigo, Palha Roxa, and São Pedro were compared to a commercial hybrid (BM207). We measured tolerance based on plant growth indices in insect-protected, relative to unprotected, plants, and in the landraces relative to the commercial hybrid. The results highlighted the potential of maize landraces showing tolerance per at least one plant parameter in comparison to the commercial hybrid BM207.

## 2. Materials and Methods

### 2.1. Experimental Conditions and Maize Genotypes

Field experiments were carried out at the Center for Scientific and Technological Development of the Lavras Federal University (UFLA), Fazenda Muquém, located in the municipality of Lavras, Minas Gerais state, Brazil (21°14′45″ S, 44°59′59″ W and 918 m asl). The experiment was replicated in the 2017/2018 (hereafter “season 1”) and 2018/2019 (“season 2”) summer cropping seasons. Sowing was carried out manually on 21 December 2017 and 15 November 2018. Field temperatures had low fluctuations in the two growing seasons, with the average maximum temperature ranging from 27.6 to 30.4 °C for season 1, and 26.9 to 30.9 °C for season 2 [50]. On the other hand, rainfall showed high variation between the seasons, being higher in season 2, with monthly accumulation from 143.6 mm (January 2019) to 323.2 mm (December 2018), while in season 1 it varied from 3.2 mm (April 2018) to 240.2 mm (January 2018) [50].

We evaluated six maize genotypes: five landraces, Amarelão, Aztequinha, Branco Antigo, Palha Roxa, and São Pedro, and the conventional (non-Bt), double hybrid, BM207 (Sementes Biomatrix^®^ Patos de Minas, Minas Gerais, Brazil), which is a genotype indicated for the south and southeast region of the country, according to the company information. The seeds of the landraces were provided by the non-governmental organization AS-PTA Farming Family and Agroecology, located in the municipality of Palmeira, Paraná State, Brazil, from the 2016 harvest. All seeds were stored in a cold chamber at 11 °C until use. This study is registered in the National System of Genetic Resource Management and Associated Traditional Knowledge (SisGen) under the code AAFDB1D.

### 2.2. Management Practices and Experimental Model

The initial preparation of the experimental site consisted of eliminating weeds by spraying the herbicide atrazine (Nortox^®^ 500 SC) at a commercial dose of 4 L ha^−1^. This was followed by fertilization with nitrogen, phosphorus, and potassium (NPK 08-28-16). The herbicide was sprayed again 30 days after sowing to ensure cleanliness of the site and between rows of maize, and manual weeding was carried out when necessary. Topdressing fertilization with urea (200 kg ha^−1^) was performed 40 days after sowing to maintain fertilization.

The experimental design was a randomized block with four replications (blocks). Each experimental plot consisted of three rows spaced 0.6 m apart and six plants per row spaced 0.25 m apart (18 plants/plot) for season 1, and eight plants per row for season 2 (24 plants/plot). Thus, the total area used for each maize genotype was 1.8 m^2^ (season 1) and 2.4 m^2^ (season 2). A spacing of 0.5 m between plots and 1.0 m between blocks was used to facilitate the evaluations.

A control block was established with the dimensions and treatment as described above, though this block was treated biweekly with the insecticide lambda-cyhalothrin (Karate-Zeon^®^ 50 CS) at the recommended dose of 150 mL ha^−1^ for control of FAW [51]. Given the winds prevailing during the experimental periods, it was inadvisable to locate control plots within each of the four replicate blocks, so the control block was located at a ~7 m distance from the nearest block to avoid insecticide drift from to insecticide-free blocks.

### 2.3. Data Collection

The experiments were carried out under natural infestation of herbivorous insects. At the growth stages V4, V6, V8, and V12 (i.e., four, six, eight, and twelve completed expanded leaves) and at the beginning of reproductive stage, we recorded the presence of relevant pest on the plots (Table 1). The FAW leaf injury was scored using rates from 0 to 9 (0 = no damage; 9 = severe damage) [52] on the youngest leaf to avoid resampling older leaves and remeasuring past injury. Additionally, we recorded the numbers of *Dalbulus maidis* (DeLong & Wolcott) (Hemiptera: Cicadellidae) because this pest was frequently found in the plots, and it is a relevant pest with economic importance in Brazil and other Latin American countries [53,54]. FAW injury and numbers of *D. maidis* were evaluated on three maize plants randomly selected in each row of the plots (9 plants/plot), and were used as covariables in statistical analyses (see below).

We evaluated three plant vegetative parameters (chlorophyll content, plant height, and stem diameter) as indices of tolerance of maize genotypes; however, chlorophyll content was measured at V6 and reproductive stage, which totalized four tolerance parameters (Table 1); growth stage V6 is the beginning of the phase of greatest growth and water consumption [55]. Chlorophyll content was measured non-destructively using the SPAD-502 meter (Konica Minolta Sensing, Tecnal, Piracicaba, São Paulo State, Brazil). The readings were carried out on the youngest expanded leaf of two plants per row (6 plants/plot), with two evaluations per plant, recording the average of the readings. Finally, plant growth was measured as plant height and stem diameter at the end of the crop cycle, from three plants per row (9 plants/plot). Height was measured as the length from the soil surface to the insertion of the last expanded leaf (m), while stem diameter (mm) was measured below the insertion of the first ear, with the aid of a digital caliper (MTX^®^). All variables were measured for the insecticide-free blocks and control (insecticide-treated) block.

### 2.4. Data Analyses 

Each of the plant variables were converted to a ratio, according to the following formula:

$$Tolerance\;ratio=\frac{Cultivar\;without\;insecticide\;}{Average\;of\;cultivar\;with\;insecticide}.$$

Data analyses consisted of analysis of variance (ANOVA) for the tolerance ratios, and included the independent variables *plant genotype* (Amarelão, Aztequinha, Branco Antigo, Palha Roxa, São Pedro, and BM207), *season* (1 and 2), and the interaction term *plant genotype × season*; additionally, FAW injury score and corn leafhopper number per plant were included as independent covariables. The ratios were normalized by converting them to their log values prior to the ANOVA. Dunnett’s post hoc test (α = 0.05) was used to compare mean ratios between each landrace and the hybrid BM207 within the main effect of plant genotype, while a priori contrasts were used to compare averages between each landrace and BM207 within the interaction effect of plant genotype × season; the critical *p* for each a priori contrast was set at 0.010 per Sidak’s correction [56]. All statistical analyses were performed using the JMP^®^ Pro 14.0.0 software [57]. 

To examine whether maize genotypes exposed to FAW displayed tolerance, i.e., compensated for tissue loss, we performed one-sample *t*-tests, with the log-transformed ratio, using the statistical software “R”, version 4.0.3 [58]. The one-sample *t*-test tested the null hypothesis that tolerance ratios did not differ from 1 (i.e., H_0_ = 1, plants exposed to FAW did not differ from plants not exposed to FAW). For interpretation of results, tolerance ratios < 1 were considered indicative of undercompensation, i.e., no tolerance, and values ≥ 1 as indicative of compensation or overcompensation, respectively (i.e., tolerance in both cases) [59]. The critical *p* for each *t*-test was set at 0.014, per the Bonferroni correction [56]. 

Finally, hierarchical clustering analysis was performed using the Ward method to group genotypes per the four tolerance ratios [57]. This analysis was conducted on per-maize genotype, average tolerance ratios for each of the plant variables. All results showing tolerance ratios are presented as back-transformed averages of the transformed values used for statistical analyses. 

## 3. Results

ANOVA revealed significant effects of maize genotype, season, and the genotype × season interaction on the tolerance ratios for all tolerance parameters, except that chlorophyll content at V6 stage was not affected by season (Table 2). The covariates FAW injury and *D. maidis* adults did not significantly affect the four tolerance parameters (Table 2). FAW injury ratio average varied from 1.24 to 4.00 in season 1, and 0.40 to 2.66 in season 2, while the average of the five evaluations of number of *D. maidis* adults varied from 0.38 to 1.93 and 0.09 to 0.69 in season 1 and 2, respectively (Tables S1 and S2). The raw mean values of the evaluation parameters are available in Tables S1 and S2.

**Figure 1:**
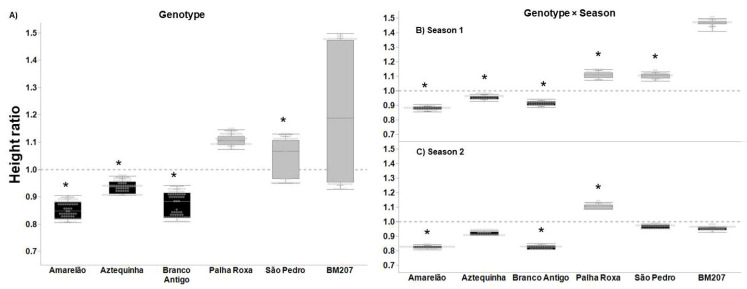
Tolerance ratio to FAW (= plant without insecticide application/average of plants with insecticide application) based on the growth parameter plant height in six maize genotypes (**A**), six genotypes in season 1 (**B**), and six genotypes in season 2 (**C**). In each plot, asterisks indicate statistical difference relative to BM207, per Dunnett’s test (**A**), and per a priori contrasts (**B**,**C**) with critical *p* ≤ 0.010 per Sidak’s correction. In each plot, black-filled boxes indicate non-tolerance (undercompensation, ratio < 1), and gray-filled boxes indicate tolerance (overcompensation, ratio > 1) (critical *p* = 0.014 per Bonferroni correction).

Hybrid BM207 and the landraces Palha Roxa and São Pedro showed overcompensation for plant height (ratio > 1.0, *p* ≤ 0.001), while Amarelão, Aztequinha, and Branco Antigo displayed undercompensation (Figure 1A, ratio < 1, *p* < 0.0001). The plant height ratio of landraces genotypes was lower than that of BM207 (*p* < 0.0001), except for Palha Roxa, which did not differ from BM207 (Figure 1A, *p* = 0.126). In season 1, BM207 showed the highest tolerance height ratio (ratio = 1.45, *p* < 0.0001), which overcompensated for FAW feeding, as well as Palha Roxa and São Pedro landraces (Figure 1B, *p* < 0.0001). In season 2, Palha Roxa was the only genotype that showed overcompensation (ratio = 1.12, *p* < 0.0001) and had a higher ratio than hybrid BM207 (Figure 1C, *p* < 0.0001, F = 16.844).

**Figure 2:**
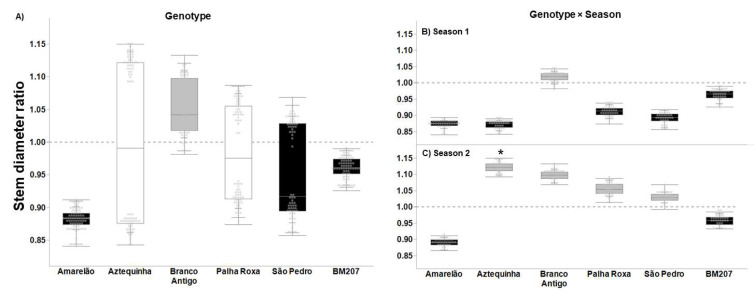
Tolerance ratio to FAW (= plant without insecticide application/average of plants with insecticide application) based on the growth parameter stem diameter in six maize genotypes (**A**), six genotypes in season 1 (**B**), and six genotypes in season 2 (**C**). In each plot, asterisks indicate statistical difference relative to BM207, per Dunnett’s test (**A**), and per a priori contrasts (**B**,**C**) with critical *p* ≤ 0.010 per Sidak’s correction. In each plot, black-filled boxes indicate non-tolerance (undercompensation, ratio < 1), gray-filled (overcompensation, ratio > 1) and white-filled (compensation, ratio = 1) boxes indicate tolerance (critical *p* = 0.014 per Bonferroni correction).

The genotypes Aztequinha, Branco Antigo, and Palha Roxa exhibited tolerance (ratio ≥ 1) according to their plant stem diameters (Figure 2A). Amarelão, São Pedro, and BM207 did not display tolerance (Figure 2A, *p* < 0.0001), and no landrace differed from hybrid BM207 (Figure 2A, *p* ≥ 0.099). In season 1, Branco Antigo was the only tolerant genotype (overcompensation (Figure 2B, ratio = 1.02, t = 7.173, *p* < 0.0001)); however, no genotypes differed from BM207 (Figure 2B, *p* ≥ 0.061). In season 2, in addition to Branco Antigo, Aztequinha, Palha Roxa, and São Pedro showed overcompensation for stem diameter (*p* < 0.0001), and the ratio in Aztequinha was greater than in BM207 (Figure 2C, F = 1.745, *p* = 0.007).

**Figure 3:**
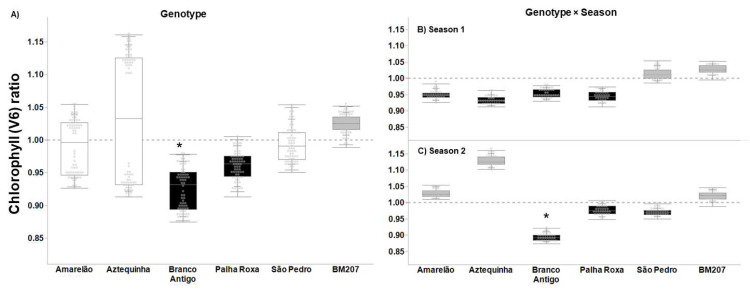
Tolerance ratio to FAW (= plant without insecticide application/average of plants with insecticide application) based on the parameter chlorophyll content at V6 stage in six maize genotypes (**A**), six genotypes in season 1 (**B**), and six genotypes in season 2 (**C**). In each plot, asterisks indicate statistical difference relative to BM207, per Dunnett’s test (**A**), and per a priori contrasts (**B**,**C**) with critical *p* ≤ 0.010 per Sidak’s correction. In each plot, black-filled boxes indicate non-tolerance (undercompensation, ratio < 1), gray-filled (overcompensation, ratio > 1) and white-filled (compensation, ratio = 1) boxes indicate tolerance (critical *p* = 0.014 per Bonferroni correction).

Genotypes Amarelão, Aztequinha, and São Pedro exhibited tolerance per their V6 stage chlorophyll ratios (ratio = 1, *p* ≥ 0.016), but not genotypes Branco Antigo and Palha Roxa (undercompensation) (Figure 3A, ratio < 1, *p* < 0.0001). BM207 showed overcompensation (*p* < 0.0001) and significantly differed from Branco Antigo (Figure 3A, *p* = 0.004). Season had no significant effect on the relative chlorophyll content in V6 plants (Table 2, *p* = 0.193). There was no significant genotype × season interaction between BM207 and the genotypes in season 1 (Figure 3B, *p* ≥ 0.044). Conversely, Branco Antigo had the lowest ratio in season 2 (Figure 3C, ratio = 0.89, F = 9.522, *p* = 0.002). Hybrid BM207 showed overcompensation in both seasons (*p* ≤ 0.0001), and São Pedro showed overcompensation in season 1 (Figure 3B, *p* < 0.0001), and Amarelão and Aztequinha in season 2 (Figure 3C, *p* < 0.0001).

**Figure 4:**
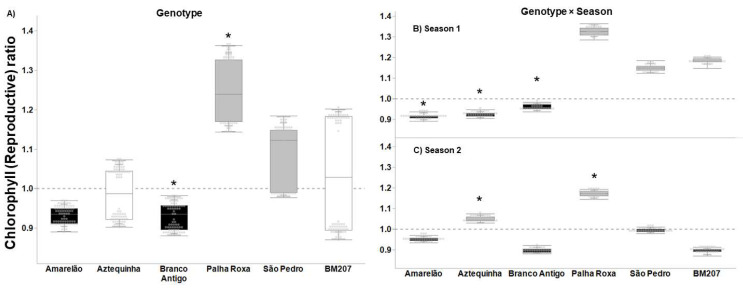
Tolerance ratio to FAW (= plant without insecticide application/average of plants with insecticide application) based on the parameter chlorophyll content at reproductive stage in six maize genotypes (**A**), six genotypes in season 1 (**B**), and six genotypes in season 2 (**C**). In each plot, asterisks indicate statistical difference relative to BM207, per Dunnett’s test (**A**), and per a priori contrasts (**B**,**C**) with critical *p* ≤ 0.010 per Sidak’s correction. In each plot, black-filled boxes indicate non-tolerance (undercompensation, ratio < 1), gray-filled (overcompensation, ratio > 1) and white-filled (compensation, ratio = 1) boxes indicate tolerance (critical *p* = 0.014 per Bonferroni correction).

The landrace Palha Roxa overcompensated for the chlorophyll content during the reproductive stage (Figure 4A, ratio = 1.25, F = 79.743, *p* < 0.0001) and showed a higher tolerance ratio than hybrid BM207 (Figure 4a, *p* < 0.0001). The genotypes Aztequinha and São Pedro were tolerant through compensation (*p* = 0.032) and overcompensation (*p* < 0.0001), respectively (Figure 4A). In the genotype × season interaction, Palha Roxa was the only genotype to show overcompensation in both seasons (Figure 4B,C, *p* < 0.0001). Amarelão, Aztequinha, and Branco Antigo were lower than BM207 in season 1 (Figure 4B, *p* = 0.0001), while Aztequinha and Palha Roxa were superior to BM207 in season 2 (Figure 4C, *p* ≤ 0.002).

**Figure 5:**
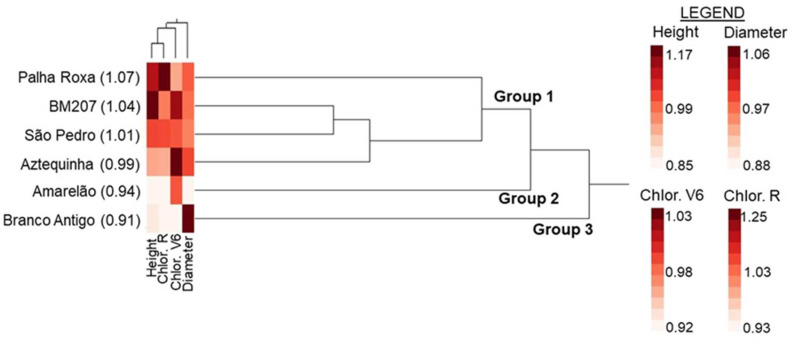
Hierarchical clustering and heat map for three FAW tolerance ratios (see text): Plant height (Height), chlorophyll content at V6 stage (Chlor. V6) and at reproductive stage (Chlor. R), and stem diameter (Diameter). The heat map shows changes (within columns) in tolerance ratios across plant genotypes (see Legend: intense color = highest tolerance, light color = lowest tolerance). Numbers following the genotypes are geometric averages of the four tolerance ratios; averages of 1.0 and above suggest tolerance, while averages below 1.0 suggest non-tolerance [59].

Hierarchical cluster analysis revealed three groups based on similarity across tolerance indices (Figure 5). The first group included Palha Roxa, BM207, São Pedro, and Aztequinha; all were considered tolerant to FAW because the geometric averages across the four tolerance ratios were ≈1.0–1.1 for each of these genotypes. The second group included only Amarelão, and the third group only Branco Antigo; both groups were considered not tolerant to FAW because their geometric averages across the four tolerance ratios were ≈0.9 for each of these genotypes (Figure 5).

## 4. Discussion

This study investigated tolerance of maize genotypes to FAW herbivory under field conditions in terms of several relevant plant growth parameters (plant height, stem diameter, and chlorophyll content at two growth stages), considering the natural factors of insect infestation, climate, and soil conditions. The presented results contribute to expanding knowledge in the literature about plant tolerance research conducted under a realistic field scenario, which is mostly scarce [60]. Here, we used tolerance indices that were calculated as the average ratio per genotype in the insecticide-free plots and the corresponding average in the insecticide-treated plot (control). The tolerance response levels were classified as undercompensation, compensation, or overcompensation when the calculated tolerance ratios were below, equal to, or above 1.0, respectively [31,59]. We discussed the results based on plant growth and chlorophyll variables related to biomass and grain yields because it was not possible to obtain these yield data in both field seasons. The maize genotypes compensated for herbivory regarding the evaluated parameters, showing some tolerance level in at least one of the four measured parameters.

Feeding injury by FAW larvae and number of corn leafhoppers did not vary among the evaluated maize genotypes, and differences in those plant parameters were not explained by variation in insects’ infestations, as demonstrated by their nonsignificant effects as covariates in the statistical model. This information is very important to point out, as the different responses in plant growth (compensation, overcompensation, and undercompensation) of genotypes were due to inherent mechanisms of tolerance, and not because of varying insect infestation and injury, which could be related to plant resistance through antixenosis and/or antibiosis [24]. Therefore, given that insect infestation and environmental conditions in the field were quite similar among maize genotypes, there is evidence that the varying responses of plant growth among genotypes were in function of intrinsic tolerance levels to insect herbivory. 

Stem diameter was a useful index for tolerance of maize genotypes. For this plant trait, landrace Branco Antigo consistently showed the highest tolerance index (overcompensation), though it was less tolerant per the other indices. Stem diameter is an important agronomic maize plant trait, as it is directly related to greater ear length and number of grains per row on the ear [61,62], as well as to the capacity to withstand environmental stresses [63]. Increases in stem diameter may be correlated with the ability to allocate more photoassimilates from damaged tissues to storage structures. The reallocation of resources, mainly carbon, is a key tolerance mechanism to leaf injury, whether natural or mechanical [64,65,66,67]. Biochemically, this plant response can be regulated by protein kinases [67] or by the induction of jasmonate derivatives, which may vary according to plant species and genotypes [66,68]. Despite having highlighted by overcompensating in landrace Branco Antigo, stem diameter might have more importance as a tolerance trait against stemborers.

Differences in plant height have also been evaluated as a tolerance response to herbivory [69,70,71]. Here, we observed that maize genotype influenced the expression of tolerance, and the genotypes Palha Roxa, São Pedro, and BM207 showed overcompensation, while the others displayed undercompensation (no tolerance). Wild cotton plants under artificial defoliation by *Spodoptera exigua* (Hübner) (Lepidoptera: Noctuidae) larvae also showed low compensation for plant height in attack levels equal to or greater than 25% [72]. On the other hand, *Schizotetranychus oryzae* Rossi de Simons (Acari: Tetranychidae) mite infestation did not affect the height of rice plants (*Oryza sativa*) [73]. The height overcompensation for the Palha Roxa, São Pedro, and BM207 genotypes may reflect an important agronomic trait because plant height correlates with increased forage crop yield [74]. As leaf herbivory by FAW can negatively affect plant growth parameters, such as height, further work is needed to assess the relationship between plant height and grain yield between genotypes infested and not infested by FAW [70]. 

Chlorophyll content was used to infer possible effects on the photosynthetic rate of maize genotypes as chlorophyll is the main pigment and positively correlates with the ability of plants to perform photosynthesis [75,76,77]. Increased photosynthetic activity is one of the main tolerance mechanisms of plants [30,31]. The tolerance ratio of chlorophyll content of the maize genotypes in our study varied according to the growth stage, which was expected, as plant age can affect several compensation parameters [78,79,80,81]. The genotypes Palha Roxa and São Pedro presented higher chlorophyll ratios during the reproductive stage, which may be related to increased photosynthetic capacity, and the higher values for this parameter coincided with those of plant height.

Tolerance is defined as the ability of plant species and genotypes to withstand or recover from herbivory caused by arthropod pests, resulting in greater biomass and/or yield compared to susceptible (non-tolerant) plants under similar pest infestation levels [24,40]. Plant capacity to compensate for herbivory is related to alterations in physiological and metabolic processes, such as increases in photosynthetic activity, antioxidant metabolism, use of stored reserves, compensatory growth, and branching [30,82,83]. In some cases, mainly upon mild herbivory by chewing insects, these tolerance mechanisms can result in overcompensation in both vegetative and reproductive plant parameters [33,81]. Stem herbivory, e.g., affects the architecture of woody plants by stimulating branch growth [83]. It is important to emphasize that tolerance mechanisms do not impose negative effects on insects’ behavior and biology, thus not exerting selection pressure on their populations and not contributing to evolution of resistance [24]. 

Generally, leaf area reduction caused by defoliation increases the photosynthetic activities in the remaining tissues [84,85], which can be explained by the “source–sink hypothesis”. According to this hypothesis, the photosynthetic rate increases with the reduction of the source supply [86]. The source comprises the tissues responsible for the acquisition and export of resources (e.g., carbon in leaves), while the sink involves the tissues responsible for its assimilation and importation (e.g., nitrogen in the leaves) [87]. The literature reports several examples that support this hypothesis [86,88,89]. Plants of *Cucumis sativus* under herbivory by *Helix aspersa* Muller (Gastropoda, Stylommatophora) showed increased photosynthetic capacity with consequent compensatory plant growth [89]. This is in line with some of our results in that greater defoliation in landraces Palha Roxa and São Pedro and hybrid BM207 in untreated plants provided plants with higher height and chlorophyll content than the insecticide-treated plants with lower injury. However, this is not a rule that applies to all herbivory situations [40,90,91]. 

Herbivory can affect plant primary growth due to changes in primary metabolism [85,92]. Primary metabolism is responsible for energy generation [93], and changes in the allocation of primary compounds can alter plant defense, growth, and reproduction mechanisms [92]. However, plant growth is a complex process that is affected by many physiological and metabolic pathways, and is mediated by oscillating levels of phytohormones and their synergistic and antagonistic crosstalk. For example, high levels of jasmonic acid, either endogenous or exogenously applied, are known to reduce plant height in rice, tobacco (*Nicotiana attenuata*), *Arabidopsis thaliana*, and maize [94,95,96,97]. One of the reasons is the inhibition of gibberellin production, a phytohormone used to regulate plant growth and development that plays an important role in stem elongation [95]. Thus, similar levels of FAW herbivory in maize genotypes may have caused distinct alterations in physiological and metabolic pathways, ultimately impacting the outcome of plant growth, with genotypes showing some levels of tolerance.

As previously mentioned, there are limited studies in the literature evaluating tolerance mechanisms in plant genotypes against insect herbivory. Among the results available, maize tolerance to *D. virgifera virgifera* increases with increasing resources availability for plant growth and reproduction, which may result in changes in metabolite and phytohormone concentrations [59,98], and improvement of stem growth (circumference and mass) because of greater carbon allocation [39]. Additionally, tolerance levels appear to be mediated by crop domestication, spread, and breeding; for example, stem diameter compensated for belowground larval injury in Mexican and US maize landraces post *D. virgifera virgifera* infestation, while Balsas teosintes and US inbred maize lines undercompensated [59]. Plant stem is a tank of photoassimilates [99], and the gain in stored reserves results in energy for growth or regrowth [85]. The reserves of photoassimilates, such as carbon and proteins, stored in the stem of tomatoes (*S. lycopersicum*) were used for leaf regrowth after complete defoliation in plants infested with larvae of *Manduca sexta* (Linnaeus) (Lepidoptera: Sphingidae) [100]. From the results obtained in our study, a suggestion of follow-up research would be investigating the relationship between variability of the growth parameters herein evaluated and the plant responses to increasing levels of FAW herbivory in order to correlate these plant traits as reliable proxies of tolerance.

Our results suggest that some of the evaluated maize genotypes are capable of compensating for FAW injury under field conditions. However, plants are subject to various biotic and abiotic variables that can affect their compensation capacity for multiple stresses. Important sources of variation include soil nutritional levels [64,101], light availability [79], abundance of herbivores [28], levels of infestation [35,36], natural enemies [102], and microorganisms [64,103]. Furthermore, the capacity of plants to compensate for injury is influenced by plant genotype, as shown in this study and others [78,101,104,105], and by other environmental conditions [106], such as rainfall, which varied between seasons in our study, as evidenced by the significant effect of growing season.

The domestication and breeding processes of maize are other variables that influence plant tolerance to herbivory, as modern hybrids and cultivars tend to allocate more resources to productivity (growth and reproduction) than to defense against herbivorous insects [59,99,107]. Tolerance of the landraces relative to the commercial hybrid varied across the measured tolerance indices in our study. The tolerance of three landraces, namely, Palha Roxa, São Pedro, and Aztequinha, were comparable to that of BM207, as suggested by the results of our cluster analysis. This is broadly consistent with expectations of comparative tolerance levels in landraces and modern maize cultivars [59,98].

Tolerance through compensation for insect herbivory without a yield tradeoff is a promising plant trait for incorporating to crop cultivars and hybrids through genetic breeding programs [107]. Our preliminary work showed that three maize landraces displayed promising levels of tolerance to FAW herbivory, compared to a commercial hybrid. In addition to serving as sources of genes conferring tolerance to FAW, these landraces can be used in sustainable production systems as an integral part of IPM strategies, as tolerant genotypes are expected to level up economic injury levels and economic thresholds, benefiting the reduction of insecticide application for pest control [29]. Future studies are needed to determine how each of the tolerance indices that we measured affects plant yield under different levels of pest infestation, their heritability, and the mechanisms by which they contribute to enhanced tolerance. This will benefit the development of a practical protocol for evaluation of tolerance in maize genotypes under field conditions that usually requires estimation of yield upon harvest at the end of crop cycle, which may be time- and labor-consuming. We highlight the need for future experiments in different locations, with larger experimental plots, and grain yield evaluation to extrapolate our results to different conditions. 

## Figures and Tables

**Table 1:** Evaluations of maize genotypes in an experimental site of the Lavras Federal University in the municipality of Lavras, Minas Gerais state, Brazil, and the respective dates.

Parameters Evaluated	DevelopmentalStage	Season/Evaluation Date	
		2017/2018	2018/2019
*Spodoptera frugiperda* leaf injury and number of *Dalbulus maidis* adults	V4	5 January	11 December
	V6	19 January	26 December
	V8	6 February	11 January
	V12	20 February	23 January
	Reproductive	21 March	12 February
Chlorophyll content	V6	19 January	26 December
	Reproductive	21 March	12 February
Plant growth	Post-reproductive	28 April	1 April

**Table 2:** Analysis of covariance (ANOVA) statistics for the independent variables genotype (Amarelão, Aztequinha, Branco Antigo, Palha Roxa, São Pedro, and BM207), season (1 and 2), and genotype × season interaction for the tolerance ratio plant height, stem diameter, and chlorophyll content at vegetative stage V6 and reproductive stage. The FAW injury and *D. maidis* was added to the model as covariates.

Source		Ratio							
	DF	PlantHeight		StemDiameter		ChlorophyllV6		Chlorophyll Reproductive	
		F	*p*	F	*p*	F	*p*	F	*p*
Genotype	5, 376	55.823	**<0.0001**	4.027	**0.001**	3.626	**0.003**	16.383	**<0.0001**
Season	1, 376	35.573	**<0.0001**	14.186	**<0.001**	1.707	0.193	7.622	**0.006**
Genotype × Season	5, 376	20.699	**<0.0001**	2.655	**0.023**	4.604	**0.001**	7.079	**<0.0001**
FAW injury	1, 376	0.015	0.902	1.403	0.237	1.568	0.212	0.418	0.519
*D. maidis*	1, 376	3.508	0.062	0.003	0.960	0.458	0.499	0.470	0.489

## Data Availability

All data is provided in the manuscript.

## Supplementary Materials

The following supporting information can be downloaded at: https://www.mdpi.com/article/10.3390/insects13070651/s1, Table S1: Mean of plant height, stem diameter, and chlorophyll content in the V6 and reproductive stage of the maize genotypes evaluated during season 1. The value of FAW injury and number of *D. maidis* is the average from the 5 evaluations in the blocks without insecticide spray; Table S2: Mean of plant height, stem diameter, and chlorophyll content in the V6 and reproductive stage of the maize genotypes evaluated during season 2. The value of FAW injury and number of *D. maidis* is the average from the 5 evaluations in the blocks without insecticide spray.
