# Supplementary material for: Comparative Tolerance Levels of Maize Landraces and a Hybrid to Natural Infestation of Fall Armyworm
Source: Insects. 2022 Jul 19;13(7):651. doi: 10.3390/insects13070651 (PMC9316814; doi:10.3390/insects13070651)
Supplement: Supplementary file 1 [file insects-13-00651-s001.zip › insects-1736359-supplementary.pdf]

Table S1. Mean of plant height, stem diameter, and chlorophyll content in the V6 and reproductive stage of the maize genotypes evaluated during season 1. The value of FAW injury and number of *D. maidis* is the average from the 5 evaluations in the blocks without insecticide spray.

Season 1							
**Block**	**Maize genotype**	**Plant height (m)**	**Stem diameter (mm)**	**Plant chorophyll V6**	**Plant chorophyll reproductive**	**FAW injury**	**Number of *D. maidis***
1	Amarelão	1.13	24.21	32.14	41.21	4.00	0.51
	Aztequinha	1.22	18.22	34.98	46.78	2.40	0.89
	Branco Antigo	1.25	22.21	35.6	44.33	2.17	0.89
	Palha Roxa	1.13	18.73	32.58	39.64	3.47	0.38
	São Pedro	1.30	19.13	33.63	40.31	1.38	0.82
	BM207	0.77	19.62	35.24	43.51	1.69	0.71
2	Amarelão	1.30	16.93	31.84	39.42	2.91	0.49
	Aztequinha	1.41	18.30	33.15	47.08	2.69	0.71
	Branco Antigo	1.37	22.05	32.73	36.22	2.82	0.93
	Palha Roxa	1.44	19.40	34.72	42.26	2.50	0.77
	São Pedro	1.46	17.35	36.5	42.99	3.24	0.73
	BM207	0.96	22.34	32.22	48.46	2.76	1.09
	Amarelão	1.41	19.55	36.43	45.79	2.96	0.84
	Aztequinha	1.35	17.61	32.12	46.69	2.09	0.42

Season 1							
**Block**	**Maize genotype**	**Plant height (m)**	**Stem diameter (mm)**	**Plant chorophyll V6**	**Plant chorophyll reproductive**	**FAW injury**	**Number of *D. maidis***
3	Branco Antigo	1.21	19.30	31.56	33.53	2.10	1.04
3	Palha Roxa	1.37	20.83	33.40	45.45	1.50	0.64
	São Pedro	1.50	17.96	32.07	39.86	2.07	0.78
	BM207	0.84	18.36	27.78	43.43	2.02	1.36
4	Amarelão	1.18	19.95	28.90	35.43	2.51	0.60
	Aztequinha	1.32	17.98	35.45	48.78	3.22	0.62
	Branco Antigo	1.32	18.55	30.95	37.44	1.40	0.91
	Palha Roxa	1.37	17.73	36.23	44.70	1.40	1.93
	São Pedro	1.21	18.89	28.57	38.50	2.00	1.07
	BM207	0.98	20.81	29.43	44.58	1.24	1.33
5 (Control Insecticide)	Amarelão	1.41	22.43	33.75	42.78	-	-
	Aztequinha	1.37	20.46	35.98	50.74	-	-
	Branco Antigo	1.40	20.02	33.95	38.84	-	-
	Palha Roxa	1.18	20.51	36.02	32.06	-	-
	São Pedro	1.23	20.25	31.79	34.52	-	-
	BM207	0.60	20.78	29.92	37.35	-	-

Table S2. Mean of plant height, stem diameter, and chlorophyll content in the V6 and reproductive stage of the maize genotypes evaluated during season 2. The value of FAW injury and number of *D. maidis* is the average from the 5 evaluations in the blocks without insecticide spray.

Season 2							
**Block**	**Maize genotype**	**Plant height (m)**	**Stem diameter (mm)**	**Plant chorophyll V6**	**Plant chorophyll reproductive**	**FAW injury**	**Number of *D. maidis***
1	Amarelão	1.56	22.75	41.83	36.51	1.40	0.29
	Aztequinha	1.63	29.26	42.86	46.12	1.33	0.09
	Branco Antigo	1.57	29.93	41.63	39.38	1.20	0.69
	Palha Roxa	1.41	30.75	36.88	31.21	2.66	0.33
	São Pedro	1.62	27.22	37.63	39.02	2.04	0.29
	BM207	1.08	26.45	43.63	45.44	1.24	0.36
2	Amarelão	1.67	9.55	41.75	36.88	1.33	0.20
	Aztequinha	1.69	15.38	45.45	41.16	1.96	0.16
	Branco Antigo	1.63	19.27	39.88	38.12	0.76	0.47
	Palha Roxa	1.78	22.13	39.47	39.32	2.11	0.16
	São Pedro	1.72	17.48	42.58	45.05	1.93	0.22
	BM207	1.07	20.31	46.10	42.68	1.69	0.44
3	Amarelão	1.72	19.85	46.99	38.17	0.77	0.33
	Aztequinha	1.64	20.93	47.58	39.2	0.84	0.18
	Branco Antigo	1.61	23.02	44.20	30.91	0.81	0.30

Season 2							
**Block**	**Maize genotype**	**Plant height (m)**	**Stem diameter (mm)**	**Plant chorophyll V6**	**Plant chorophyll reproductive**	**FAW injury**	**Number of *D. maidis***
3	Palha Roxa	1.68	21.64	40.78	37.22	2.21	0.51
	São Pedro	1.56	16.21	43.00	39.63	0.87	0.31
	BM207	1.13	20.83	43.88	44.09	0.53	0.42
4	Amarelão	1.66	20.08	44.34	39.00	0.78	0.53
	Aztequinha	1.72	17.85	42.18	40.10	0.40	0.40
	Branco Antigo	1.40	23.02	38.08	35.69	1.35	0.18
	Palha Roxa	1.81	19.35	42.62	37.81	1.16	0.26
	São Pedro	1.70	18.50	42.96	42.73	1.89	0.40
	BM207	1.27	18.77	44.98	44.53	0.64	0.60
5 (Control Insecticide)	Amarelão	2.00	18.86	42.25	39.96	-	-
	Aztequinha	1.81	17.80	39.13	39.28	-	-
	Branco Antigo	1.87	20.74	45.63	40.33	-	-
	Palha Roxa	1.57	20,49	40.45	30.89	-	-
	São Pedro	1.69	18.36	41.99	41.36	-	-
	BM207	1.18	22.06	43.48	49.02	-	-
